# Mapping Brain–Computer Interface Research in Depression (1995–2026): A Bibliometric Analysis

**DOI:** 10.1002/brb3.71896

**Published:** 2026-11-03

**Authors:** Yicheng Jiang, Xianlun Duan

**Affiliations:** ^1^ Boston University's College of Engineering Boston Massachusetts USA; ^2^ Department of Thoracic Surgery Anhui Provincial Children's Hospital Hefei China

**Keywords:** bibliometric analysis, brain–computer interface, depression, emotion regulation, neuromodulation

## Abstract

**Introduction:**

Brain–computer interfaces (BCIs) have emerged as promising tools for neuromodulation and emotion regulation in depression. This study aimed to systematically map the global research landscape, thematic evolution, and emerging trends of BCI applications in depressive disorders through bibliometric analysis.

**Methods:**

Data were retrieved from the Web of Science Core Collection and analyzed using the R package bibliometrix, VOSviewer, and CiteSpace. Publication trends, country and institutional contributions, journal impact, author influence, and keyword‐based clustering and burst analysis were comprehensively evaluated.

**Results:**

A total of 239 publications authored by 1284 researchers from 239 institutions across 39 countries were identified. The United States and China had the highest publication outputs, while the United Kingdom showed high citation impact. Laureate Institute for Brain Research was the most productive institution and showed the strongest institutional collaboration, while *NeuroImage: Clinical* showed the highest journal h‐index. Masaya Misaki was the leading author according to publication and citation indicators. Keyword analysis identified six major thematic clusters involving neurophysiological and cognitive features, EEG asymmetry and affective regulation, clinical neurofeedback, connectivity and self‐regulation, fMRI‐based emotion regulation, and psychiatric symptom/network correlates. “Emotion” showed the strongest keyword burst, while recent bursts in “real‐time fMRI neurofeedback,” “prefrontal cortex,” and “emotion regulation” indicated increasing research attention to circuit‐level regulation.

**Conclusion:**

The field has evolved from EEG‐based characterization toward network‐level and real‐time neurofeedback research. These findings identify emerging research directions while highlighting the need for further methodological standardization and clinical validation.

## Graphical abstract

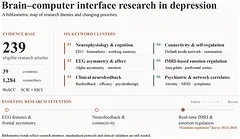

## 1 Introduction

Brain–computer interfaces (BCIs) are emerging neurotechnologies that acquire and process neural activity to generate outputs for communication, control, assessment, or modulation (Chaudhary et al. 2016). Neurofeedback is a related closed‐loop neurotechnology in which processed neural signals are returned to participants to facilitate the self‐regulation of brain activity (Sitaram et al. 2017). Although neurofeedback is not identical to a conventional BCI controlling an external device, both approaches involve the acquisition and processing of neural signals to produce a functionally meaningful output. Accordingly, this study adopted a broader but explicitly defined analytical framework encompassing conventional BCI applications and neurofeedback‐based approaches relevant to depression research.

Depression is one of the most prevalent mental health disorders globally. A systematic review and meta‐analysis reported a pooled prevalence of depression of 32.0% among the populations studied during the COVID‐19 pandemic (Shao et al. 2024). A global burden analysis reported an increase in the prevalence and burden of depressive disorders during the COVID‐19 pandemic, with younger age groups showing greater increases in prevalence (Santomauro et al. 2021). This alarming rise underscores the importance of developing effective interventions for treatment‐resistant depression, particularly as traditional therapeutic options are insufficient for many patients.

Over the past two decades, research at the intersection of BCI and mental health has grown substantially, driven by advances in neurotechnology, signal processing, and machine learning. BCI systems, particularly those based on electroencephalography (EEG), have been investigated for their potential in decoding affective states and providing neurofeedback or modulated neural stimulation tailored to depressive symptomatology (Khorev et al. 2024). A recent review discussed the clinical applications of BCI technologies, including their potential use in depression assessment and treatment (Jia et al. 2025). Moreover, randomized controlled trials of real‐time functional magnetic resonance imaging (fMRI) neurofeedback have been conducted in major depressive disorder (MDD), signaling a shift toward translational applications (Mehler et al. 2018). However, the rapid proliferation of studies at the intersection of neuroscience, engineering, and psychiatry has resulted in a fragmented body of knowledge. The interdisciplinary nature of this field, spanning everything from signal processing algorithms to clinical psychiatric protocols, makes it increasingly difficult for researchers to grasp the overall trajectory of research, identify key areas of consensus, and distinguish exploratory findings from clinically validated evidence. Consequently, the existing literature must be systematically organized.

Bibliometric analysis offers a robust method to systematically map the scientific landscape, identify influential research themes, quantify publication trends, and reveal collaborative networks in this evolving field. While bibliometric studies have been conducted in related domains, for example, EEG‐based BCI research in rehabilitation medicine (Angulo Medina et al. 2024), there is a distinct gap in comprehensive bibliometric evaluation targeting the application of BCI technologies in depression research. The present study was therefore conducted to map BCI applications in depressive disorders and to identify key contributors, thematic focuses, and emerging trends in the existing literature.

## 2 Materials and Methods

### 2.1 Search Strategies and Data Collection

We searched the Web of Science Core Collection (WoSCC), specifically the Science Citation Index Expanded (SCIE) and Social Sciences Citation Index (SSCI), for BCI and neurofeedback literature related to depression. The WoSCC was selected for its rigorous journal screening and standardized citation indexing, making it a reliable, widely used database for bibliometric analysis. The search formula was: TS = (“brain‐computer interface*” OR “brain computer interface*” OR “brain‐machine interface*” OR “brain machine interface*” OR “mind‐machine interface*” OR “mind machine interface*” OR “neurofeedback” OR “neuro‐feedback” OR “EEG neurofeedback” OR “EEG biofeedback” OR “fMRI neurofeedback” OR “real‐time fMRI neurofeedback” OR “real time fMRI neurofeedback” OR “rtfMRI neurofeedback”) AND (AB = (“depression” OR “major depressive disorder*” OR “major depression” OR “depressive symptom*” OR “treatment‐resistant depression” OR “treatment resistant depression” OR “unipolar depression” OR “MDD”) OR TI = (“depression” OR “major depressive disorder*” OR “major depression” OR “depressive symptom*” OR “treatment‐resistant depression” OR “treatment resistant depression” OR “unipolar depression” OR “MDD”)).

For eligibility assessment, BCI‐related studies were operationally defined as meeting at least one of two criteria: (i) studies explicitly framed as BCI or brain–machine interface research in which neural signals were acquired and computationally decoded or classified to generate an application‐relevant output in the context of depression or (ii) neurofeedback studies in which processed neural activity was returned to participants in real time or near real time to facilitate self‐regulation. Neurofeedback was therefore included as a related closed‐loop neurotechnology rather than treated as synonymous with conventional BCI. General EEG or neuroimaging studies, open‐loop neuromodulation studies, and neural‐association or classification studies were excluded when they lacked either an explicitly identified BCI application or a closed‐loop feedback process.

The final search was conducted on August 20, 2026. Records not classified as “Article” and non‐English‐language records were excluded. The remaining records were independently screened by two reviewers for substantive relevance to both depression and BCI/neurofeedback. A publication was eligible when depression or a depressive disorder constituted the primary study population, clinical condition, therapeutic target, prespecified primary outcome, or principal analytical focus and when BCI or neurofeedback represented a substantive methodological or intervention component according to the operational definition above. Studies of adjacent conditions, including post‐stroke depression, were retained only when depression met one of these criteria; neurological rehabilitation studies that mentioned depressive symptoms only incidentally were excluded. Disagreements were resolved through discussion until consensus was reached.

### 2.2 Statistical Analysis

Bibliometric analyses were performed using VOSviewer 1.6.20, CiteSpace 6.4.R1, and bibliometrix 4.6.1. VOSviewer was used to construct country, institutional, and author co‐authorship networks; journal co‐citation and bibliographic‐coupling networks; and the keyword co‐occurrence network. Full counting and association‐strength normalization were applied, with other clustering parameters retained at their default settings. The minimum thresholds were two publications for journals and countries/regions, three for institutions and authors, and four occurrences for keywords. Bibliometrix was used to calculate descriptive and citation‐based indicators (Derviş 2019).

CiteSpace analyses covered January 1995 to August 2026 using 1‐year time slices, with “Keywords” as the node type and the top five keywords per slice as the selection criterion (Top N = 5). Pathfinder pruning and pruning of merged networks were applied. Keyword burst detection used the same temporal coverage and cleaned keyword dataset (Synnestvedt et al. 2005). Before analysis, author names were disambiguated using full names, ResearcherID/ORCID records, affiliations, research topics, and co‐authorship patterns, while institutional variants were standardized. Keyword abbreviations, full forms, singular/plural forms, capitalization, spelling, and hyphenation variants were harmonized using a predefined thesaurus, and generic terms were removed. The h‐index, g‐index, and m‐index were calculated from the final dataset using bibliometrix: the h‐index represents h publications with at least h citations each, the g‐index gives greater weight to highly cited publications, and the m‐index adjusts the h‐index for the number of years since the author's or journal's first publication in the dataset (Bertoli‐Barsotti and Lando 2017; Bornmann et al. 2008; Egghe 2006). Journal impact factors and quartiles were obtained from the 2025 Journal Citation Reports (JCR); these indicators were interpreted as measures of bibliometric productivity and citation prominence rather than scientific quality or clinical relevance.

## 3 Results

### 3.1 Publication Landscape and Temporal Trends

A bibliometric analysis of BCI research in depression, covering the period from 1995 to 2026, initially identified 414 records. After excluding non‐English records and non‐article publication types, 277 records remained, and 38 records were further removed during manual relevance screening, leaving 239 eligible articles, as illustrated in the flowchart in Figure 1. These 239 articles were authored by 1284 researchers affiliated with 239 institutions across 39 countries/regions. The publications appeared in 142 distinct journals and cited a total of 10,933 references (Figure 2A). The number of publications increased substantially over the study period, with a notable acceleration in the late 2010s and the highest annual output observed in 2025 (Figure 2B).

**FIGURE 1:**
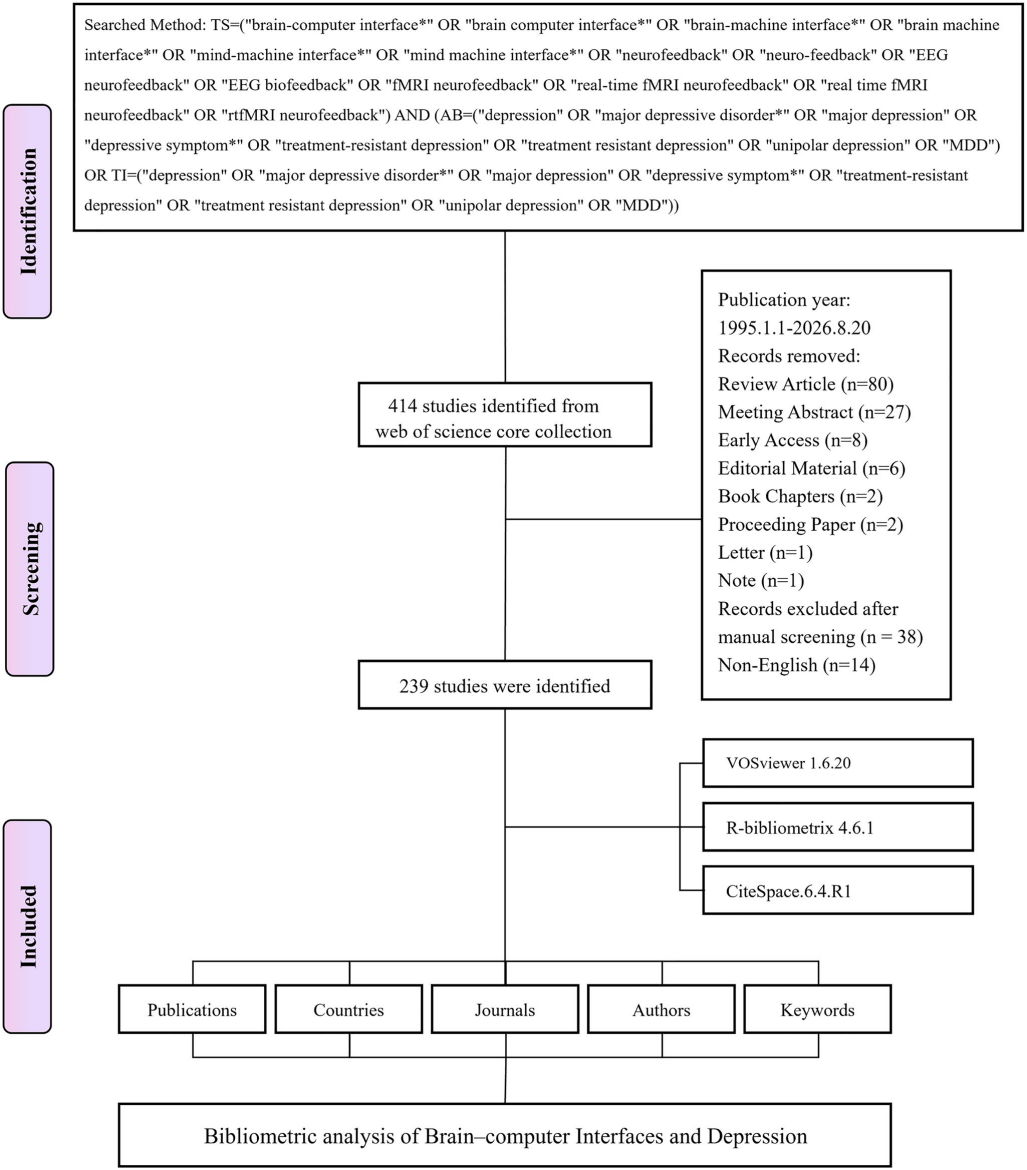
Flowchart of literature identification, screening, and inclusion for the bibliometric analysis of brain–computer interface research in depression. A total of 414 records were retrieved from the Web of Science Core Collection. After exclusion based on publication type, language, and manual relevance screening, 239 English‐language articles were included in the final bibliometric analysis. The included records were analyzed using VOSviewer (version 1.6.20), R‐bibliometrix (version 4.6.1), and CiteSpace (version 6.4.R1).

**FIGURE 2:**
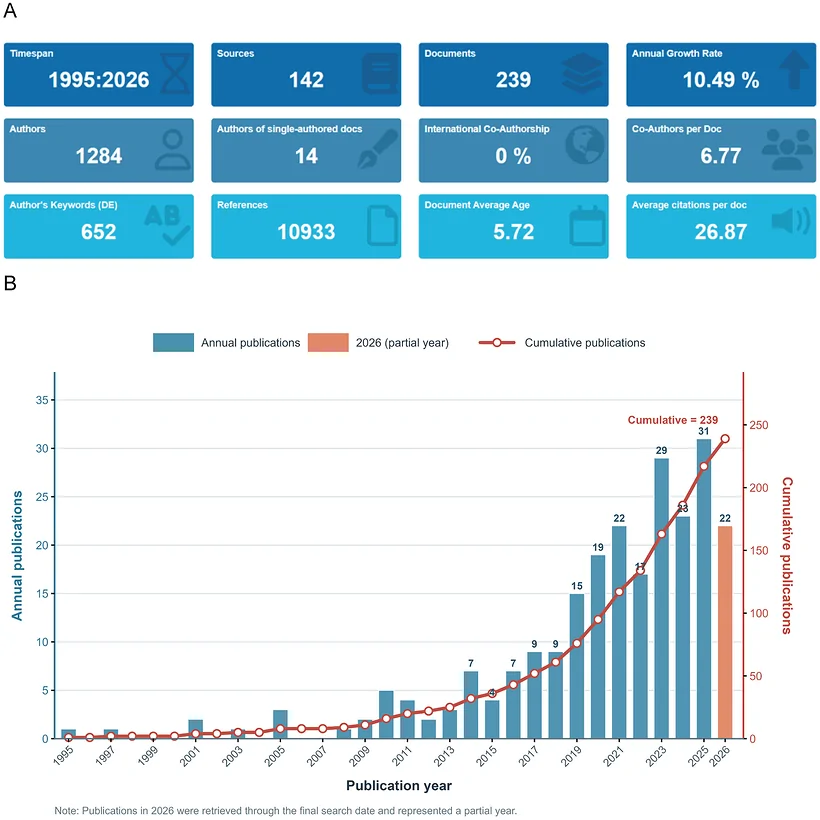
Overview of the included dataset and annual publication trend of brain–computer interface research in depression from 1995 to 2026. (A) Summary of the main bibliometric characteristics of the final dataset, including the timespan, number of sources, documents, authors, institutions, countries/regions, author keywords, references, annual growth rate, and average citations per document. (B) Annual and cumulative number of publications included in the final dataset. Publication data for 2026 represent records indexed up to August 20, 2026, and therefore correspond to a partial publication year.

### 3.2 Most Cited Publications

The most frequently cited publication in the final dataset was “Real‐Time Self‐Regulation of Emotion Networks in Patients with Depression,” published in *PLOS One* (2024 impact factor [IF] = 2.9) in 2012, with 285 citations. This study introduced a real‐time fMRI neurofeedback approach that enabled patients with depression to regulate emotion‐related brain networks (Linden et al. 2012). The second most cited publication was “Randomized Clinical Trial of Real‐Time fMRI Amygdala Neurofeedback for Major Depressive Disorder: Effects on Symptoms and Autobiographical Memory Recall,” published in the *American Journal of Psychiatry* (2024 IF = 15.1) in 2017, with 242 citations (Young et al. 2017). The third most cited publication was “Real‐time fMRI neurofeedback training of amygdala activity in patients with major depressive disorder,” published in *PLOS One* in 2014, with 206 citations (Young et al. 2014). These highly cited studies collectively demonstrate the prominent role of real‐time fMRI neurofeedback and emotion‐related neural regulation in the depression‐focused literature.

### 3.3 Impact of Journal

The 10 most influential journals, ranked by total citations, are displayed in Table S1. *NeuroImage: Clinical* had the highest h‐index (9), with a g‐index of 10, an m‐index of 0.818, 10 publications, and 488 citations. *Journal of Affective Disorders* had an h‐index of 7 and the highest g‐index (11) and m‐index (0.875) among the listed journals, with 11 publications and 202 citations, while *Applied Psychophysiology* and *Biofeedback* had an h‐index of 7, a g‐index of 9, and 9 publications. The co‐citation journal network included 38 journals with at least two publications. The journals with the highest total link strength were *NeuroImage: Clinical* (51), *PLOS One* (45), and *Journal of Affective Disorders* (34) (Figure 3A). The bibliographic coupling network also included 38 journals, with *NeuroImage: Clinical* (1413), *Journal of Affective Disorders* (944), and *Scientific Report*s (543) showing the highest total link strengths (Figure 3B).

**FIGURE 3:**
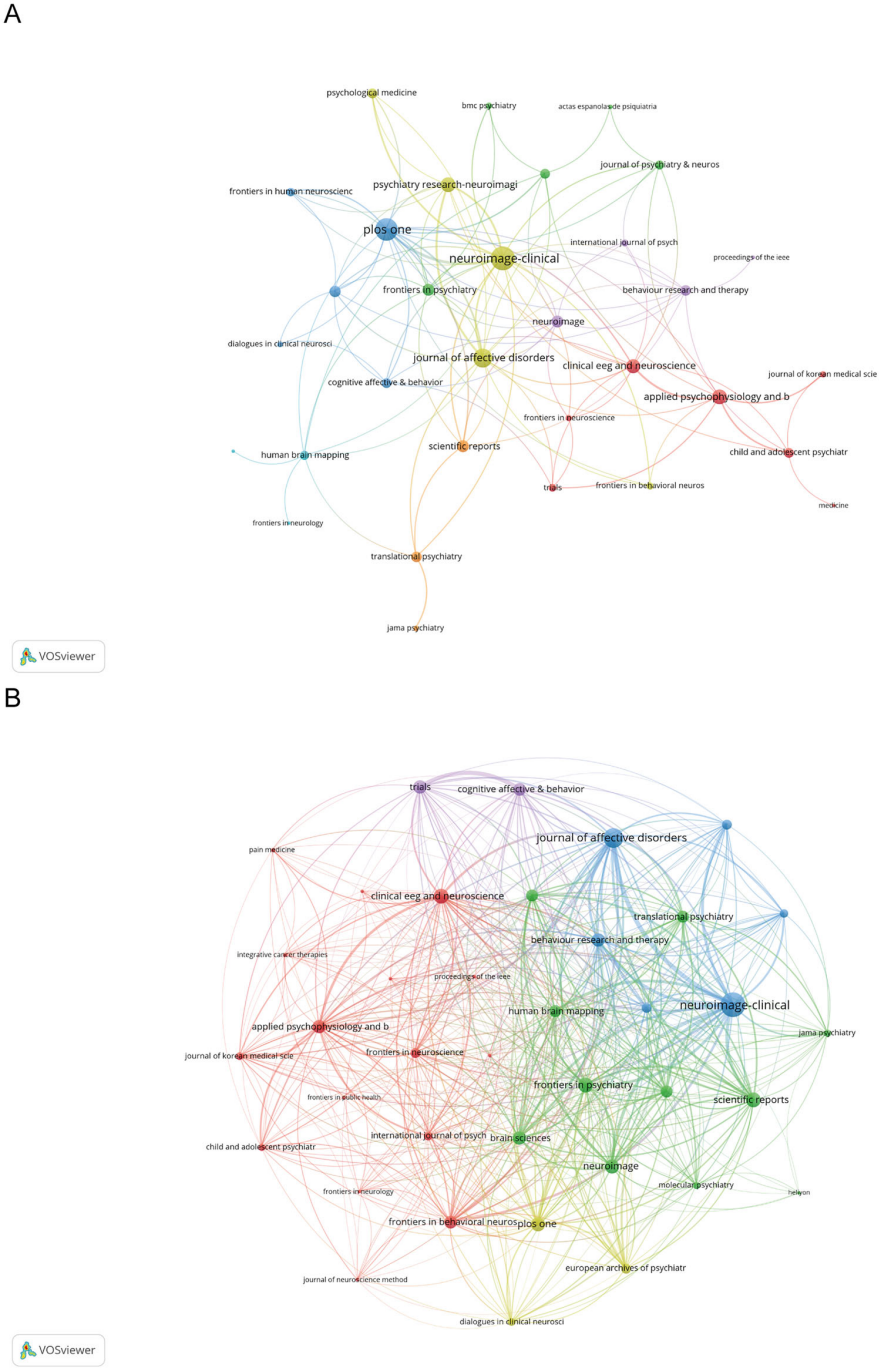
Journal network analysis of brain–computer interface research in depression. (A) VOSviewer‐based journal co‐citation network comprising 38 journals. Links indicate that publications from two journals were cited together in the same source documents, link thickness reflects co‐citation strength, and node colors indicate clusters. (B) Journal bibliographic‐coupling network including journals with at least two publications. Links indicate shared references between journals, and greater total link strength indicates a stronger shared intellectual basis.

### 3.4 Global Contributions and Collaborative Strengths

Based on the corresponding author's country, the top 20 countries accounted for 224 articles, representing 93.7% of the dataset. The USA ranked first with 57 articles, followed by China with 38 and the United Kingdom with 13 (Figure 4A). Table S2 reports the publication and citation indicators separately: the United States had 77 total publications (TPs) and 2600 total citations (TCs), while China had 42 TPs and 558 TCs. The United Kingdom ranked second in TCs (1475), with an average of 50.9 citations per publication. Regarding internationally co‐authored publications, Germany and the United States each had nine multiple‐country publications (MCPs), followed by China and the United Kingdom with eight each (Figure 4A). The country collaboration network included 32 countries with at least two publications. The United States showed the strongest international collaboration, with a total link strength of 43, followed by the United Kingdom (34) and Germany (24) (Figure 4B).

**FIGURE 4:**
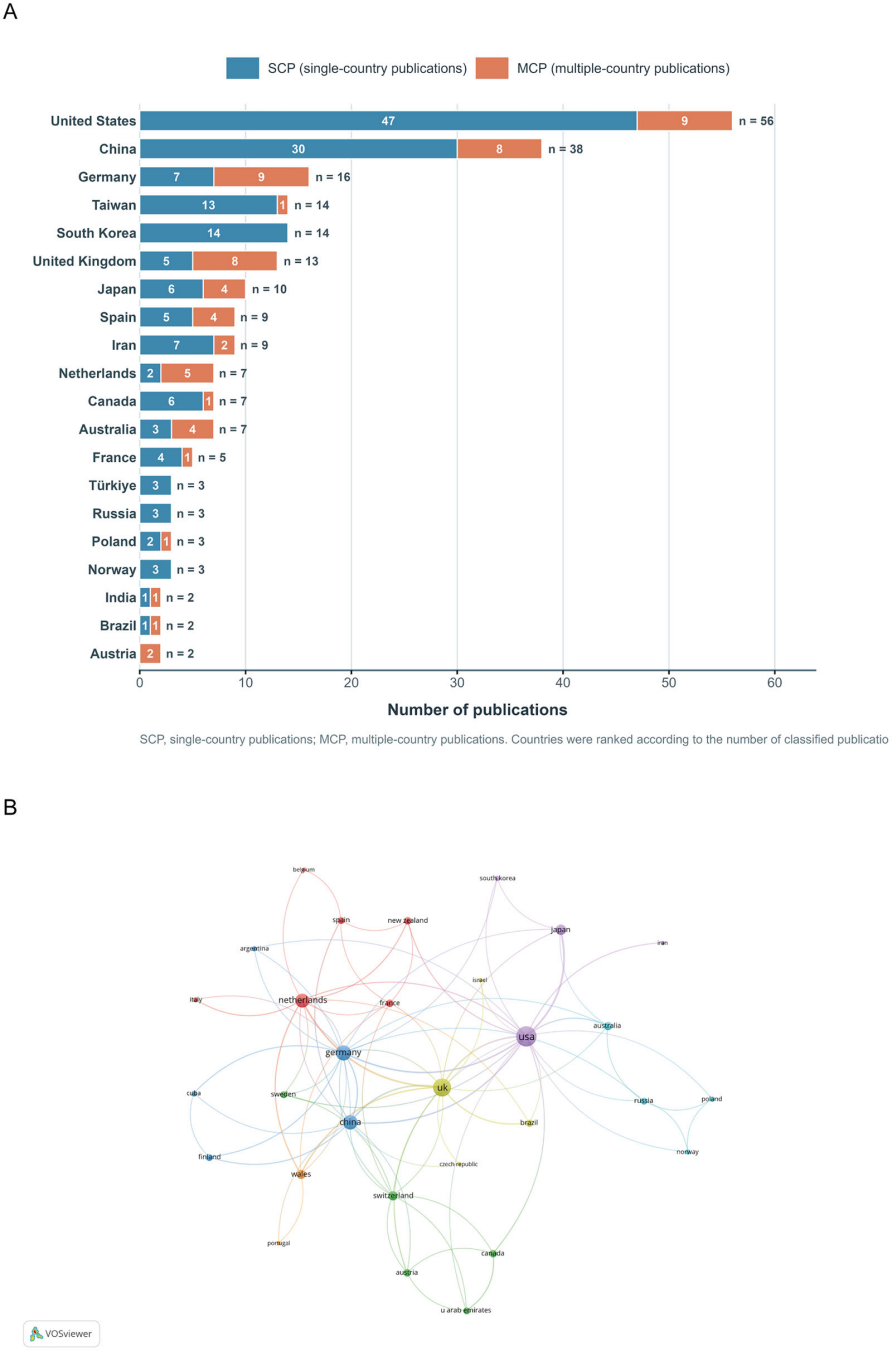
Country distribution and international collaboration in brain–computer interface research in depression. (A) Publications according to the corresponding author's country. Blue segments represent single‐country publications (SCP), and orange segments represent multiple‐country publications (MCP), defined as publications involving international co‐authorship. (B) VOSviewer‐based collaboration network including 32 countries/regions with at least two publications. Node size represents publication output, links indicate intercountry collaboration, link thickness reflects collaboration strength, and node colors indicate collaboration clusters.

### 3.5 Institutional Footprint: Productivity and Partnerships

The top 10 institutions ranked by publication output are shown in Figure 5A. The Laureate Institute for Brain Research ranked first with 16 publications, followed by the University of Pittsburgh with 14 publications. Harvard University and the University of Oklahoma–Norman each contributed 11 publications, while Kaohsiung Medical University and King's College London each contributed eight. The displayed institutional collaboration network comprised 20 connected institutions with at least three publications. The Laureate Institute for Brain Research showed the highest total link strength (33), followed by Kyoto University and Showa University, both with a total link strength of 30 (Figure 5B).

**FIGURE 5:**
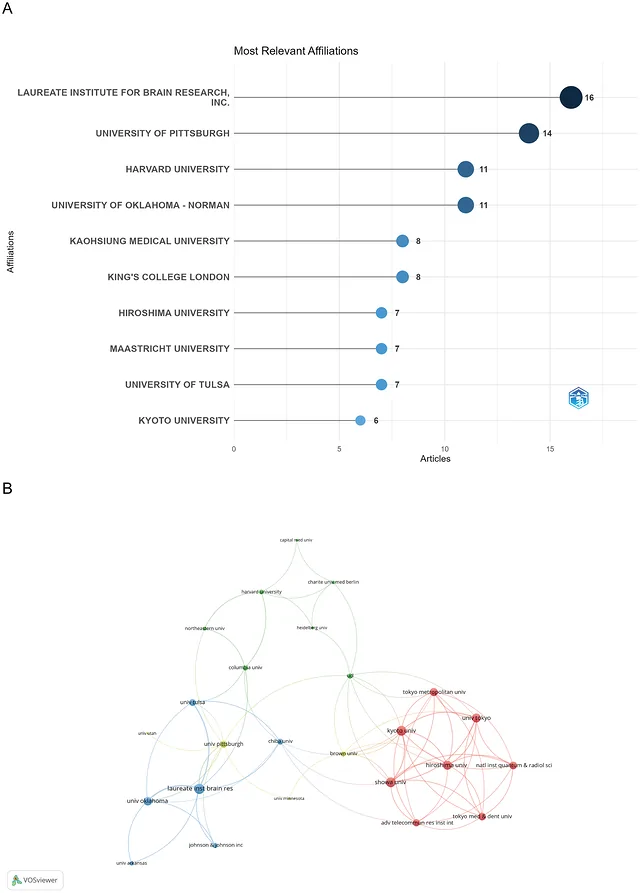
Institutional productivity and collaboration in brain–computer interface research in depression. (A) Top 10 institutions ranked by publication output; circle size represents the number of publications, and darker shading indicates a higher rank. (B) VOSviewer‐based collaboration network including institutions with at least three publications. Node size represents publication output, links indicate institutional collaboration, link thickness reflects collaboration strength, and node colors indicate collaboration clusters.

### 3.6 Author Impact and Network of Collaboration

The 20 authors with the highest corpus‐level h‐indices are presented in Table S3. Masaya Misaki ranked first in publication output (TPs = 14), h‐index (12), g‐index (14), and TC (998), followed by Jerzy Bodurka and Vadim Zotev. Their respective m‐indices were 0.92, 0.85, and 0.77. Among the 53 authors with at least three publications included in the collaboration network, Masaya Misaki showed the highest collaboration strength (54), followed by Yasumasa Okamoto (50) and Jerzy Bodurka (45) (Figure 6).

**FIGURE 6:**
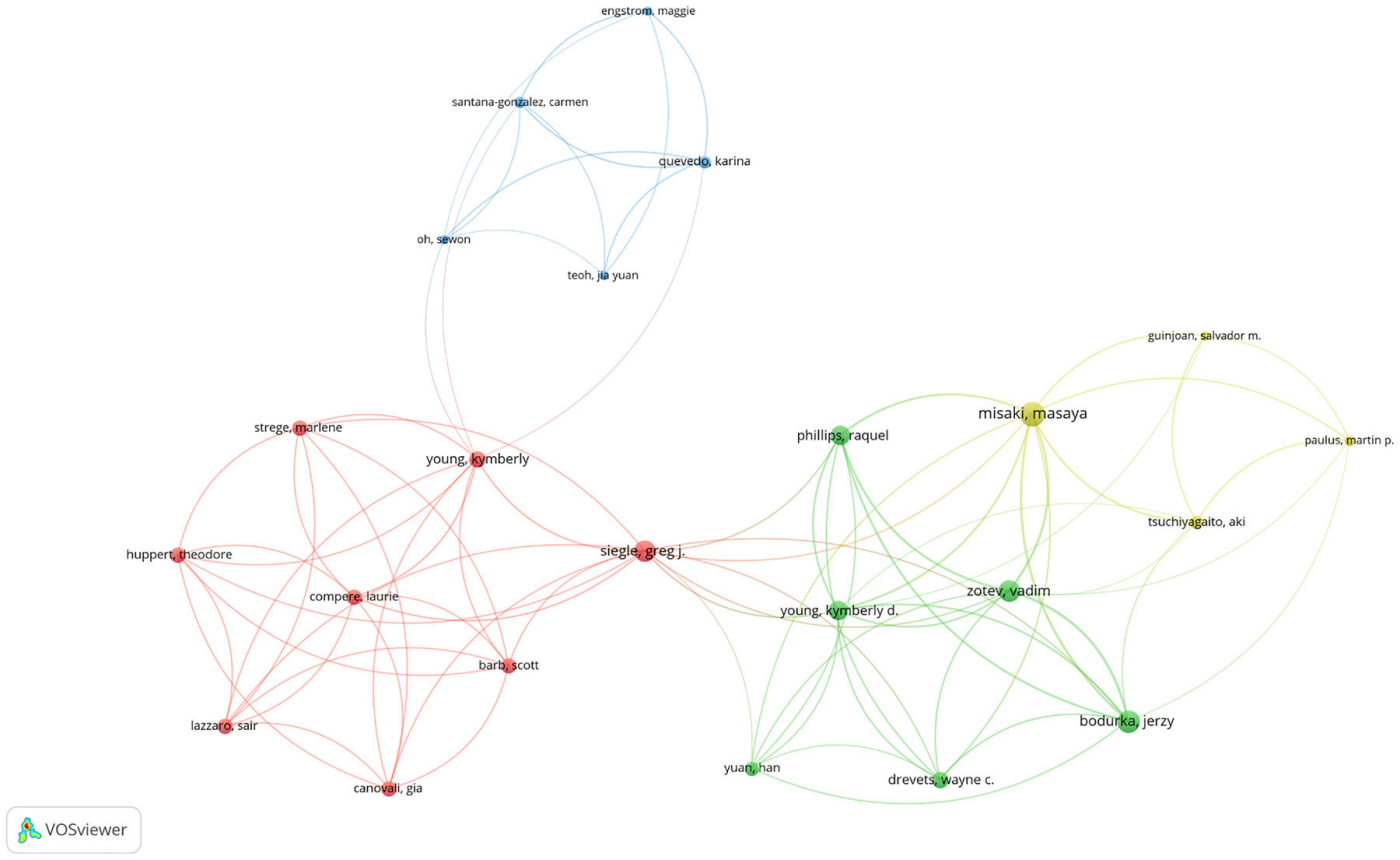
Author collaboration network in brain–computer interface research in depression. The VOSviewer network includes authors with at least three publications. Node size represents publication output, links indicate co‐authorship, link thickness reflects collaboration strength, and node colors indicate author collaboration clusters.

### 3.7 Analysis of the Keywords

#### 3.7.1 Keyword Cluster Analysis

A comprehensive keyword co‐occurrence network analysis was performed using the VOSviewer application. In total, 76 keywords with a minimum of four occurrences were identified. “Anxiety” showed the highest total link strength (TLS = 97; 34 occurrences), followed by “depression” (TLS = 89; 33 occurrences), “neurofeedback” (TLS = 77; 23 occurrences), and “EEG” (TLS = 70; 25 occurrences).

Additionally, the keyword co‐occurrence analysis revealed six major clusters, each represented by a distinct color in the visualization map. Cluster 1 mainly reflected neurophysiological and cognitive features, represented by “EEG,” “neurofeedback,” “attention,” “biomarker,” and “working memory”. Cluster 2 focused on EEG asymmetry and affective processes, with representative terms including “alpha asymmetry,” “brain asymmetry,” “EEG neurofeedback,” “emotion,” and “frontal EEG asymmetry”. Cluster 3 was related to clinical neurofeedback and therapeutic evaluation, characterized by “biofeedback,” “depression,” “efficacy,” “mental health,” and “psychotherapy”. Cluster 4 emphasized connectivity and self‐regulation approaches, including “connectivity,” “default mode network,” “real‐time fMRI neurofeedback,” “rumination,” and “self‐regulation”. Cluster 5 centered on fMRI‐based emotion regulation, represented by “amygdala,” “emotion regulation,” “functional connectivity,” “prefrontal cortex,” and “real‐time fMRI”. Cluster 6 reflected psychiatric symptoms and cortical/network correlates, including “anxiety,” “major depressive disorder,” “orbitofrontal cortex,” “state functional connectivity,” and “symptoms” (Figure 7A).

**FIGURE 7:**
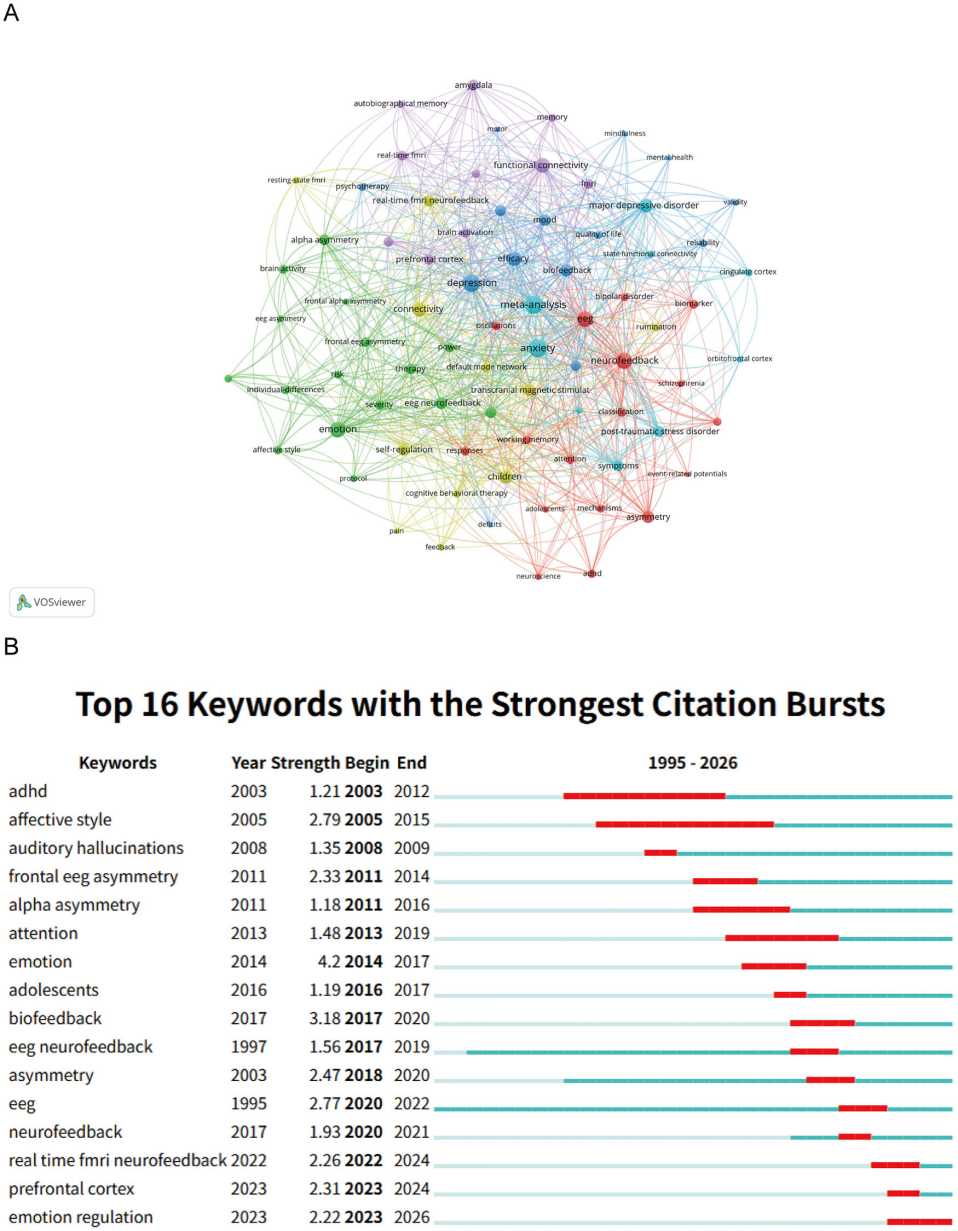
Keyword co‐occurrence and burst analysis of brain–computer interface research in depression. (A) VOSviewer‐based keyword co‐occurrence network constructed from the cleaned keyword dataset; 76 keywords with at least four occurrences were included. Node size represents keyword frequency, links indicate co‐occurrence, link thickness reflects association strength, and node colors represent the six keyword clusters. (B) CiteSpace‐based burst analysis identifying the 16 keywords with the strongest keyword bursts during 1995–2026. Red segments indicate periods of significantly increased research attention.

#### 3.7.2 Analysis of Burst Keywords

Keyword burst analysis was performed for 1995–2026 to examine changes in research attention. The 16 identified burst keywords had strengths ranging from 1.18 to 4.20. The strongest burst was observed for “emotion” (strength = 4.2; 2014–2017), followed by “biofeedback” (strength = 3.18; 2017–2020), “affective style” (strength = 2.79; 2005–2015), “EEG” (strength = 2.77; 2020–2022), “asymmetry” (strength = 2.47; 2018–2020), and “frontal EEG asymmetry” (strength = 2.33; 2011–2014). Earlier bursts were mainly related to EEG‐based affective markers and asymmetry, whereas more recent bursts shifted toward real‐time neurofeedback and emotion‐regulation mechanisms. Notably, “real‐time fMRI neurofeedback” showed a recent burst from 2022 to 2024 (strength = 2.26), while “prefrontal cortex” emerged from 2023 to 2024 (strength = 2.31). “Emotion regulation” showed a sustained burst from 2023 through 2026 (strength = 2.22), making it the only burst keyword in the current analysis that remained active through 2026 (Figure 7B).

## 4 Discussion

This bibliometric analysis provides an updated overview of BCI and neurofeedback research in depression from 1995 to 2026. The final dataset included 239 publications from 39 countries/regions, involving 1284 authors and 239 institutions, with a marked concentration of research activity in the United States, China, and the United Kingdom. The keyword analysis identified six interconnected thematic clusters, while burst analysis showed a temporal shift from EEG‐related concepts and affective asymmetry toward biofeedback, real‐time fMRI neurofeedback, prefrontal targets, and emotion regulation.

### 4.1 General Information

Among the core journals, NeuroImage: Clinical, Journal of Affective Disorders, and PLOS One occupied prominent positions in the journal co‐citation network and the bibliographic coupling network, indicating their importance in disseminating research at the intersection of neuroimaging, neurofeedback, and depression. In particular, NeuroImage: Clinical has published influential work linking frontal EEG asymmetry with amygdala activity during real‐time fMRI neurofeedback and studies combining simultaneous EEG and fMRI neurofeedback in patients with MDD (Zotev et al. 2016, 2020). These studies illustrate how the field has increasingly integrated electrophysiological and hemodynamic signals to investigate emotion regulation in depression.

At the country level, the United States remained the largest contributor, while China ranked second in publication output, and the United Kingdom showed a comparatively high citation impact. Rather than interpreting these bibliometric differences as direct indicators of scientific quality, they more conservatively reflect differences in publication volume, citation accumulation, and international research participation. At the institutional level, the Laureate Institute for Brain Research ranked first in both publication output and total collaboration strength, indicating that institutional productivity and collaborative connectivity were concentrated in the same leading center in this dataset. The University of Pittsburgh ranked second in publication output.

The author analysis further highlights the sustained contribution of Masaya Misaki, Jerzy Bodurka, and Vadim Zotev, who ranked among the leading authors in publication output, citation indicators, and collaboration strength. Their work has been particularly influential in developing amygdala‐targeted real‐time fMRI neurofeedback for MDD. Early proof‐of‐concept work demonstrated that patients with depression could learn to upregulate amygdala responses during positive autobiographical memory recall (Young et al. 2014), while a subsequent randomized controlled trial reported reductions in depressive symptoms and improvements in autobiographical memory following amygdala neurofeedback (Young et al. 2017).

### 4.2 Research Hotspots and Trends

The bibliometric analysis of BCI research in depression from 1995 to 2026 reveals distinct thematic clusters that highlight specific research foci and trends within the field.

#### 4.2.1 Cluster 1: Neurophysiological and Cognitive Features

The first cluster appears to have formed around the frequent co‐occurrence of EEG, neurofeedback, attention, biomarkers, and cognitive measures. This pattern suggests that a substantial portion of the literature has been organized around the identification of measurable neural features that can be decoded or modified through BCI/neurofeedback systems. The coexistence of diagnostic and cognitive terms within the same cluster is consistent with studies that use EEG not only to characterize abnormal neural activity but also to derive features for classification, cognitive assessment, or feedback training. EEG‐based neurofeedback studies in MDD have similarly concentrated on identifying reproducible neural targets while examining associated cognitive or clinical changes (Oprea et al. 2026). Thus, the emergence of this cluster likely reflects the convergence of signal characterization and neurofeedback‐oriented intervention design within the same research stream.

#### 4.2.2 Cluster 2: EEG Asymmetry and Affective Regulation

The second cluster is characterized by strong links among alpha asymmetry, brain asymmetry, EEG neurofeedback, emotion, and frontal EEG asymmetry. Its formation likely reflects the historical development of depression‐related EEG research, in which frontal asymmetry became both a candidate marker of affective processing and a potential target for neurofeedback. Because the same studies may simultaneously measure asymmetry, emotional state, and training‐related changes, these terms are naturally connected within the co‐occurrence network. Earlier neurofeedback research specifically used frontal EEG asymmetry to investigate whether affective patterns could be modified through self‐regulation (Mennella et al. 2017). The persistence of this cluster therefore appears to reflect a methodological lineage from electrophysiological characterization to EEG‐based self‐regulation, rather than simply the independent popularity of asymmetry‐related terms.

#### 4.2.3 Cluster 3: Clinical Neurofeedback and Therapeutic Evaluation

The third cluster brings together biofeedback, depression, efficacy, mental health, and psychotherapy, suggesting that neurofeedback research has increasingly been connected with clinical evaluation and treatment‐oriented terminology. The appearance of these terms in the same thematic group can be interpreted as evidence that studies in this stream are not limited to neural signal manipulation but increasingly relate training paradigms to symptom outcomes and conventional psychological treatment. This interpretation is consistent with systematic reviews showing that neurofeedback studies in MDD have examined depressive symptoms alongside different training protocols and control conditions (Dobbins et al. 2023). At the same time, the cluster should not be interpreted as evidence that efficacy has been established because substantial heterogeneity in protocols, sample sizes, outcome measures, and study quality remains a recurring limitation (Melnikov 2021).

#### 4.2.4 Cluster 4: Connectivity, Self‐Regulation, and Network‐Based Approaches

The fourth cluster links connectivity, the default mode network, real‐time fMRI neurofeedback, rumination, and self‐regulation, indicating the emergence of a research stream centered on network‐level rather than isolated regional measures. These keywords are likely grouped because studies using real‐time fMRI increasingly define intervention targets in terms of interactions among brain regions and relate those changes to maladaptive cognitive processes such as rumination. For example, connectivity‐based neurofeedback studies have directly targeted network interactions associated with repetitive negative thinking in MDD (Tsuchiyagaito et al. 2023). The clustering therefore suggests a conceptual transition from measuring individual neural features to manipulating distributed functional relationships, which may explain why connectivity and self‐regulation are structurally linked in the current knowledge map.

#### 4.2.5 Cluster 5: FMRI‐Based Emotion Regulation

The fifth cluster is organized around amygdala, emotion regulation, functional connectivity, prefrontal cortex, and real‐time fMRI. Its emergence likely reflects a relatively coherent experimental paradigm in which researchers repeatedly examine the interaction between specific affective regions and broader regulatory networks during neurofeedback training. Amygdala‐targeted real‐time fMRI studies in MDD have used positive autobiographical memory and emotion regulation tasks to examine whether patients can learn to control activity within affect‐related circuits (Young et al. 2014). A subsequent randomized trial evaluated changes in depressive symptoms and autobiographical memory (Young et al. 2017). The resulting cluster can therefore be understood as a coherent methodological and conceptual research stream linking neural targets, regulatory tasks, and real‐time fMRI‐based feedback.

#### 4.2.6 Cluster 6: Psychiatric Symptoms and Cortical/Network Correlates

The association of anxiety, major depressive disorder, orbitofrontal cortex, state functional connectivity, and symptoms suggests a thematic stream focused on connecting heterogeneous clinical phenotypes with measurable neural‐network characteristics. Anxiety frequently overlaps with depressive symptomatology, while orbitofrontal and cingulate systems are implicated in affective regulation, reward processing, and symptom expression, providing a plausible basis for their repeated co‐occurrence in the same literature. Large‐scale neuroimaging studies have demonstrated associations between orbitofrontal connectivity and depressive symptom dimensions, while other work has identified different functional‐connectivity patterns in MDD according to anxiety‐related clinical characteristics (Cheng et al. 2016; Li et al. 2025). The presence of psychometric and symptom‐related terms within this cluster further suggests an increasing interest in using neural‐network measures as intermediate phenotypes for clinically heterogeneous depression. Thus, this cluster links neural characterization with the clinical heterogeneity of depression.

#### 4.2.7 Burst Analysis

Research on BCI and neurofeedback in depression has undergone several stages of thematic development from 1995 to 2026. Early bursts centered on “affective style,” “frontal EEG asymmetry,” and related asymmetry concepts, suggesting that the field was initially shaped by efforts to characterize affective functioning through measurable electrophysiological features. Frontal asymmetry was particularly suitable for repeated measurement and later incorporation into neurofeedback paradigms (Allen et al. 2017). Thus, these early bursts appear to mark the establishment of an electrophysiological foundation for the field.

The strongest bursts, observed for “emotion” and “biofeedback,” indicate a shift from observation toward active modulation. Neural and physiological signals increasingly became not only markers but also feedback targets for self‐regulation. However, systematic evidence continues to show substantial variation in training targets, protocols, and control conditions (Melnikov 2021). The burst therefore reflects increasing intervention‐oriented research activity rather than established therapeutic efficacy.

Subsequent bursts of “EEG” and “asymmetry” suggest that signal‐level methodology remained central as the field diversified. This persistence may reflect ongoing efforts to obtain neural signals sufficiently stable for repeated online feedback, particularly given the effects of artifacts, electrode conditions, preprocessing, and signal variability (Ronca, Capotorto, et al. 2024). These findings highlight the continued relevance of EEG methods, for which reliable signal acquisition and processing are essential.

The emergence of “real‐time fMRI neurofeedback” suggests a further methodological transition toward anatomically and functionally targeted regulation. Real‐time fMRI enables feedback based on specific brain regions or functional relationships and has been applied to amygdala activity, functional connectivity, and repetitive negative thinking in depression (Tsuchiyagaito et al. 2023; González Méndez et al. 2022). The later burst of “prefrontal cortex” is consistent with increasing interest in anatomically informed regulation and circuit‐level mechanisms.

Finally, the sustained burst of “emotion regulation” represents a higher order concept that integrates EEG asymmetry, amygdala modulation, prefrontal control, and functional connectivity. This suggests that emotion regulation has become an important organizing concept connecting previously separate research streams (Zotev et al. 2020). Overall, the burst trajectory suggests a shift from electrophysiological characterization and biofeedback‐based modulation toward real‐time circuit regulation and emotion regulation. Importantly, these bursts indicate changing research priorities rather than a linear progression toward clinically validated interventions; current evidence remains limited by small samples, heterogeneous protocols, and insufficient replication (González Méndez et al. 2022).

### 4.3 Biosignal Processing and Real‐World Implementation

The increasing prominence of EEG‐related research highlights the importance of robust biosignal processing for real‐world BCI implementation. EEG is sensitive to ocular, muscular, motion‐related, and environmental artifacts, while electrode movement and signal variability can further reduce reliability outside controlled laboratory settings. Wearable EEG research therefore emphasizes robust artifact correction and preservation of neural information under realistic conditions (Ronca, Capotorto, et al. 2024). Multi‐stage ocular artifact correction algorithms developed specifically for out‐of‐the‐laboratory recordings illustrate one practical route toward this goal (Ronca, Flumeri, et al. 2024). Future depression‐related BCI studies should prioritize signal‐quality monitoring, artifact‐robust preprocessing, adaptive calibration, cross‐session validation, and computationally efficient real‐time processing.

### 4.4 Critical Appraisal and Clinical Translation

Despite increasing research activity, the evidence base remains heterogeneous in modality, feedback target, training duration, control condition, and outcome measure. Recent reviews of EEG neurofeedback and real‐time fMRI neurofeedback have highlighted small clinical samples, limited replication, and substantial protocol variation (Oprea et al. 2026; González Méndez et al. 2022). Standardized clinical outcomes, target‐engagement measures, multicenter trials, and longer term follow‐up are therefore needed. Importantly, citation counts, keyword centrality, and burst strength indicate scholarly attention rather than therapeutic efficacy or superiority.

### 4.5 Strengths and Limitations

Our study has several strengths. First, it provides an updated bibliometric overview of global research on BCI in depression from 1995 to 2026. Second, it provides comprehensive insights into top‐performing countries, authors, institutions, and journals. Several limitations should be acknowledged. First, using only the WoSCC may have excluded relevant studies indexed in other databases. Second, restricting the analysis to English‐language publications may have introduced language bias. Third, citation counts may be influenced by publication age and citation accumulation, resulting in time‐related bias. Fourth, manual cleaning of keywords and institutional names may still involve some degree of subjective judgment. Finally, bibliometric indicators reflect research attention and citation influence rather than direct evidence of clinical efficacy or scientific quality. As a bibliometric study, this analysis did not appraise the risk of bias of individual studies and did not pool treatment effects, so it cannot be used to draw conclusions about the efficacy of BCI or neurofeedback in depression.

## 5 Conclusion

This bibliometric study mapped the global research landscape and thematic evolution of BCI studies in depression from 1995 to 2026. The analysis identified major contributors, collaboration networks, core journals, and six interconnected thematic clusters, revealing a transition from EEG‐based characterization and affective asymmetry toward biofeedback, functional connectivity, real‐time fMRI neurofeedback, and emotion regulation. Recent keyword bursts further highlighted increasing research attention to prefrontal and circuit‐level regulation. Future studies should prioritize standardized neurofeedback protocols, robust biosignal processing, adequately powered multicenter trials, consistent clinical outcomes, and validation under realistic conditions to determine the clinical applicability of BCI‐based approaches for depression.

## Author Contributions

**Xianlun Duan** and **Yicheng Jiang**: conception and design. Xianlun Duan: administrative support. Yicheng Jiang: data analysis and interpretation. Xianlun Duan and Yicheng Jiang: manuscript writing. Xianlun Duan and Yicheng Jiang: final approval of manuscript.

## Funding

The authors have nothing to report.

## Conflicts of Interest

The authors declare no conflicts of interest.

## Supporting information




**Supplementary Table 1**. Bibliometric indicators of high‐impact journals in brain–computer interface research in depression. Journals are characterized using the h‐index, g‐index, m‐index, total publications (TP), total citations (TC), publication start year (PY_start), 2024 Journal Impact Factor (JIF), and 2024 Journal Citation Reports (JCR) quartile. The h‐index represents the number h of publications that have each received at least h citations; the g‐index gives greater weight to highly cited publications; and the m‐index is calculated as the h‐index divided by the number of years since the first publication within the analyzed dataset.
**Supplementary Table 2**. Publication and citation profiles of leading countries in brain–computer interface research in depression. The table summarizes publication output, single‐country publications (SCP), multiple‐country publications (MCP), MCP ratio, total publications (TP), total citations (TC), corresponding ranks, and average citations per publication for the leading contributing countries/regions. SCP represents publications authored within a single country, whereas MCP represents internationally co‐authored publications.
**Supplementary Table 3**. Publication and citation profiles of high‐impact authors in brain–computer interface research in depression. The table summarizes the h‐index, g‐index, m‐index, first publication year (PY_start), total publications (TP), fractionalized publication count (TP_Frac), publication rank, total citations (TC), and citation rank for leading authors. The h‐index represents the number h of an author's publications that have each received at least h citations; the g‐index gives greater weight to highly cited publications; and the m‐index adjusts the h‐index according to the number of years since the author's first publication within the analyzed dataset.

## Data Availability

All data generated or analyzed during this study are included in this published article.
