# Supplementary material for: Mapping Brain–Computer Interface Research in Depression (1995–2026): A Bibliometric Analysis
Source: Brain Behav. 2026 Nov 3;16(11):e71896. doi: 10.1002/brb3.71896 (PMC13729829; doi:10.1002/brb3.71896)
Supplement: Supplementary file 1 [file BRB3-16-e71896-s001.docx]

**Supplementary Table 1. Bibliometric indicators of high-impact journals in brain–computer interface research in depression.**

| **Journal** | **H-index** | **IF 2024** | **JCR 2025** | **G-index** | **M-index** | **TP** | **TP_rank** | **TC** | **TC_rank** | **PY_start** |
| --- | --- | --- | --- | --- | --- | --- | --- | --- | --- | --- |
| PLOS ONE | 4 | 2.9 | Q1 | 4 | 0.267 | 4 | 10 | 547 | 1 | 2012 |
| NeuroImage: Clinical | 9 | 4.2 | Q1 | 10 | 0.818 | 10 | 2 | 488 | 2 | 2016 |
| Frontiers in Neuroscience | 5 | 4.0 | Q2 | 7 | 0.294 | 7 | 5 | 447 | 3 | 2010 |
| Applied Psychophysiology and Biofeedback | 7 | 3.0 | Q2 | 9 | 0.269 | 9 | 3 | 351 | 4 | 2001 |
| American Journal of Psychiatry | 1 | 15.9 | Q1 | 1 | 0.100 | 1 | 39 | 242 | 5 | 2017 |
| Nature Communications | 1 | 18.1 | Q1 | 1 | 0.091 | 1 | 39 | 229 | 6 | 2016 |
| Journal of Affective Disorders | 7 | 5.7 | Q1 | 11 | 0.875 | 11 | 1 | 202 | 7 | 2019 |
| Child and Adolescent Psychiatric Clinics of North America | 2 | 2.3 | Q2 | 2 | 0.091 | 2 | 16 | 171 | 8 | 2005 |
| Behaviour Research and Therapy | 4 | 4.2 | Q1 | 4 | 0.400 | 4 | 10 | 144 | 9 | 2017 |
| Sensors | 1 | 3.4 | Q2 | 1 | 0.100 | 1 | 39 | 128 | 10 | 2017 |

Note(s): H-index: The h-index of the journal, which measures both the productivity and citation impact of the publications. IF 2024: Impact Factor, indicating the average number of citations to recent articles published in the journal. JCR 2024: The quartile ranking of the journal in the Journal Citation Reports, indicating the journal's ranking relative to others in the same field (Q1: top 25%, Q2: 25%-50%, Q3: 50%-75%, Q4: bottom 25%). G-index: The g-index of the journal, which gives more weight to highly cited articles. M-index: The h-index of the journal divided by the number of years since the journal's first publication within the analysed dataset. TP: Total Publications. TP_rank: Rank of Total Publications. TC: Total Citations. TC_rank: Rank of Total Citations. PY_start: Year of the journal's first publication within the analysed dataset. IF and JCR quartile are from the 2024 Journal Citation Reports (Clarivate). H-, G- and M-indices were calculated from the final analysed dataset of 239 articles using bibliometrix; they reflect bibliometric productivity and citation prominence, not scientific quality or clinical relevance.

**Supplementary Table 2. Publication and citation profiles of leading countries in brain–computer interface research in depression.**

| **Country** | **Articles** | **Freq** | **SCP** | **MCP** | **MCP_Ratio** | **TP** | **TP_rank** | **TC** | **TC_rank** | **Average Citations** |
| --- | --- | --- | --- | --- | --- | --- | --- | --- | --- | --- |
| United States | 57 | 0.238 | 48 | 9 | 0.158 | 77 | 1 | 2600 | 1 | 33.8 |
| China | 38 | 0.159 | 30 | 8 | 0.211 | 42 | 2 | 558 | 4 | 13.3 |
| Germany | 16 | 0.067 | 7 | 9 | 0.562 | 22 | 4 | 460 | 7 | 20.9 |
| Taiwan | 14 | 0.059 | 13 | 1 | 0.071 | 18 | 5 | 490 | 6 | 27.2 |
| South Korea | 14 | 0.059 | 14 | 0 | 0.000 | 15 | 6 | 278 | 9 | 18.5 |
| United Kingdom | 13 | 0.054 | 5 | 8 | 0.615 | 29 | 3 | 1475 | 2 | 50.9 |
| Japan | 10 | 0.042 | 6 | 4 | 0.400 | 14 | 8 | 534 | 5 | 38.1 |
| Iran | 9 | 0.038 | 7 | 2 | 0.222 | 9 | 9 | 139 | 12 | 15.4 |
| Spain | 9 | 0.038 | 5 | 4 | 0.444 | 9 | 9 | 238 | 11 | 26.4 |
| Netherlands | 7 | 0.029 | 2 | 5 | 0.714 | 15 | 6 | 949 | 3 | 63.3 |
| Canada | 7 | 0.029 | 6 | 1 | 0.143 | 9 | 9 | 116 | 13 | 12.9 |
| Australia | 7 | 0.029 | 3 | 4 | 0.571 | 8 | 12 | 70 | 21 | 8.8 |
| France | 5 | 0.021 | 4 | 1 | 0.200 | 7 | 13 | 261 | 10 | 37.3 |
| Russia | 3 | 0.013 | 3 | 0 | 0.000 | 6 | 14 | 98 | 17 | 16.3 |
| Norway | 3 | 0.013 | 3 | 0 | 0.000 | 4 | 16 | 52 | 23 | 13.0 |
| Poland | 3 | 0.013 | 2 | 1 | 0.333 | 3 | 20 | 17 | 28 | 5.7 |
| Türkiye | 3 | 0.013 | 3 | 0 | 0.000 | 3 | 20 | 114 | 14 | 38.0 |
| Switzerland | 2 | 0.008 | 1 | 1 | 0.500 | 6 | 14 | 76 | 20 | 12.7 |
| Brazil | 2 | 0.008 | 1 | 1 | 0.500 | 4 | 16 | 83 | 19 | 20.8 |
| Italy | 2 | 0.008 | 2 | 0 | 0.000 | 4 | 16 | 322 | 8 | 80.5 |

Note(s): Articles: Publications of Corresponding Authors only. Freq: Frequency of Total Publications. MCP_Ratio: Proportion of Multiple Country Publications. TP: Total Publications. TP_rank: Rank of Total Publications. TC: Total Citations. TC_rank: Rank of Total Citations. Average Citations: The average number of citations per publication.

**Supplementary Table 3. Publication and citation profiles of high-impact authors in brain–computer interface research in depression.**

| **Authors** | **H-index** | **G-index** | **M-index** | **PY_start** | **TP** | **TP_Frac** | **TP_rank** | **TC** | **TC_rank** |
| --- | --- | --- | --- | --- | --- | --- | --- | --- | --- |
| Misaki Masaya | 12 | 14 | 0.92 | 2014 | 14 | 2.20 | 1 | 998 | 1 |
| Bodurka Jerzy | 11 | 11 | 0.85 | 2014 | 11 | 1.96 | 2 | 977 | 2 |
| Zotev Vadim | 10 | 10 | 0.77 | 2014 | 10 | 1.76 | 3 | 960 | 3 |
| Siegle Greg J. | 8 | 8 | 0.80 | 2017 | 8 | 1.17 | 4 | 452 | 9 |
| Phillips Raquel | 7 | 7 | 0.54 | 2014 | 7 | 0.91 | 7 | 838 | 4 |
| Lin I-Mei | 6 | 7 | 0.75 | 2019 | 7 | 1.49 | 5 | 144 | 23 |
| Okamoto Yasumasa | 6 | 7 | 0.55 | 2016 | 7 | 0.54 | 6 | 416 | 10 |
| Young Kymberly D. | 6 | 7 | 0.46 | 2014 | 7 | 0.99 | 8 | 745 | 5 |
| Linden David E. J. | 5 | 5 | 0.26 | 2008 | 5 | 0.30 | 12 | 538 | 8 |
| Ichikawa Naho | 4 | 5 | 0.57 | 2020 | 5 | 1.03 | 10 | 167 | 21 |
| Kawato Mitsuo | 4 | 5 | 0.36 | 2016 | 5 | 0.36 | 11 | 403 | 11 |
| Quevedo Karina | 4 | 5 | 0.50 | 2019 | 5 | 2.69 | 13 | 63 | 32 |
| Yamawaki Shigeto | 4 | 4 | 0.57 | 2020 | 4 | 0.46 | 26 | 113 | 25 |
| Canovali Gia | 3 | 3 | 0.75 | 2023 | 3 | 0.49 | 31 | 36 | 45 |
| Engstrom Maggie | 3 | 4 | 0.25 | 2015 | 4 | 0.34 | 33 | 72 | 29 |
| Goebel Rainer | 3 | 3 | 0.43 | 2020 | 3 | 0.34 | 34 | 38 | 42 |
| Guinjoan Salvador M. | 3 | 3 | 0.20 | 2012 | 3 | 0.37 | 35 | 400 | 13 |
| Hashimoto Ryuichiro | 3 | 3 | 0.75 | 2023 | 3 | 0.33 | 36 | 43 | 41 |
| Hu Bin | 3 | 3 | 0.27 | 2016 | 3 | 0.92 | 18 | 303 | 18 |
| Huppert Theodore | 3 | 4 | 0.18 | 2010 | 4 | 0.39 | 37 | 117 | 24 |

Note(s): H-index: The h-index of the author, which measures both the productivity and citation impact of the publications. G-index: The g-index of the author, which gives more weight to highly cited articles. M-index: The m-index of the author, which is the h-index divided by the number of years since the first published paper. TP: Total Publications. TP_rank: Rank of Total Publications. TC: Total Citations. TC_rank: Rank of Total Citations. PY_start: Publication Year Start, indicating the year of the author's first publication within the analysed dataset. H-, G- and M-indices were calculated from the final analysed dataset of 239 articles using bibliometrix; they reflect bibliometric productivity and citation prominence, not scientific quality or clinical relevance.
